# Shape and Size Variations in the Astragalus of Large and Small Bovids

**DOI:** 10.3390/ani15030425

**Published:** 2025-02-03

**Authors:** Burak Ünal, Barış Can Güzel, Buket Çakar, Yeşim Aslan Kanmaz, Funda Yiğit, Ozan Gündemir, Mihaela-Claudia Spataru

**Affiliations:** 1 Department of Anatomy, Faculty of Veterinary Medicine, Istanbul University-Cerrahpasa, 34320 Istanbul, Türkiye; burak.unal@iuc.edu.tr; 2 Department of Anatomy, Faculty of Veterinary Medicine, Siirt University, 56100 Siirt, Türkiye; bcguzel@hotmail.com; 3 Institute of Graduate Studies, Istanbul University-Cerrahpasa, 34320 Istanbul, Türkiye; buket.cakar@ogr.iuc.edu.tr; 4 Department of Anatomy, Faculty of Veterinary Medicine, University of Fırat, 23200 Elazıg, Türkiye; yesim.aslan@firat.edu.tr; 5 Department of Histology and Embryology, Faculty of Veterinary Medicine, Istanbul University-Cerrahpasa, 34320 Istanbul, Türkiye; 6 Department of Public Health, Faculty of Veterinary Medicine, “Ion Ionescu de la Brad” Iasi University of Life Sciences, 700489 Iasi, Romania; mspatarufmv@yahoo.com

**Keywords:** geometric morphometrics, shape analysis, talus, taxonomy, veterinary anatomy

## Abstract

The astragalus acts as a crucial intermediary in the tarsal joint, facilitating the transfer of body weight to the lower extremities while also ensuring both stability and mobility through its articulation with adjacent bones, enabling efficient movement. This study investigates the morphological diversity of the astragalus in three modern cattle breeds, one goat breed, and three sheep breeds using three-dimensional geometric morphometric methods. A total of 225 specimens were analyzed in this study. Large bovids, such as the Holstein and Simmental cattle breeds, had a more developed lateral edge of the proximal trochlea and a more rounded astragalus. In small bovids, the Akkaraman and Morkaraman sheep breeds displayed a more prominent medial edge and a rounded astragalus form, while the Hair goat breed exhibited a rectangular structure with a well-developed lateral projection. The results of this study demonstrate that the astragalus serves as a reliable morphological marker for differentiating between large and small bovids, as well as between closely related taxa such as sheep and goats. These shape variations, particularly observed in the proximal trochlea, reflect functional adaptations to size, body mass, and locomotor demands, making the astragalus a critical element for taxonomic identification and biomechanical analysis.

## Simple Summary

In this study, shape variations in the astragalus, an important bone of the talus joint, in large and small bovids were investigated. By examining the shape and size of this bone in three cattle breeds, three sheep breeds, and one goat breed, we found clear differences between large animals like cattle and smaller animals like sheep and goats. Cattle had a more rounded and robust astragalus, while sheep and goats showed a narrower and more rectangular structure. Among sheep and goat breeds, the goat had a more developed outer edge, likely linked to its ability to climb rough and steep terrains. These differences are valuable for veterinary anatomy as they provide insights into bone structure and function, while also serving as important markers for identifying species and understanding taxonomic relationships among bovids.

## 1. Introduction

The astragalus (*os talus*) is a component of the tarsal joint. This bone articulates (*facies articulares calcaneae*) with calcaneus and plays a critical role in transmitting the body weight to the foot. In bovids, the astragalus shows two trochleae: the proximal trochlea (*trochlea tali proximalis*), which articulates with the tibia and malleolar bone, and the distal trochlea (*trochlea tali distalis*), which joins with the central tarsal bone and the tarsal bone IV [1,2]. Among all appendicular skeletal elements, the astragalus has limited growth potential, which occurs solely through appositional growth along the medio-lateral axis [3,4]. Unlike the skull and other bones, the astragalus is less influenced by factors such as nutritional levels and sexual dimorphism [5]. As a vital component of the ankle joint, this bone serves as a pivotal point for transforming movement both distally and dorsally [3]. Working in conjunction with the calcaneus, the astragalus regulates the degree of flexibility and stability within the joint. Additionally, the astragalus significantly impacts the morphology of the distal joint and the shape of the plantar articular surface [6].

Geometric morphometrics (GMM) is a powerful and increasingly prominent method for analyzing morphological diversity, which arises due to various biological processes such as adaptation, mutation, and genetic development [7,8]. Unlike traditional morphometric approaches that rely on linear measurements or ratios, GMM captures the geometric properties of shapes by analyzing Cartesian coordinates of homologous anatomical landmarks [9,10]. These landmarks are scaled to the same size, aligned, and superimposed using methods such as Procrustes analysis, which removes the effects of size, position, and orientation, isolating shape as the primary focus of the analysis [11,12,13]. This process enables the quantification of shape variation and facilitates the identification of vectorial changes that are the most significant for distinguishing forms.

GMM is an effective method for analyzing the complex and multidimensional morphological characteristics of the astragalus, which are difficult to quantify using traditional measurements. Due to their robust structure, astragali tend to preserve their morphology for longer periods compared to other skeletal elements, making them valuable in archaeological studies. For instance, Haruda et al. [3] successfully distinguished astragali from different sheep populations across various regions of Central Asia. Similarly, Pöllath et al. [5] employed GMM to analyze astragali from prehistoric contexts in Southwest Asia, including the Early Neolithic, Middle Chalcolithic, and Late Bronze Age, revealing shape variations between wild and domesticated sheep breeds. In another study, Colominas et al. [14] explored size and shape variability in sheep astragali, examining samples spanning from the Middle Iron Age to the early Roman period. Modern reference studies on bones can further increase the accuracy and interpretation of such analyses, providing important comparative data for understanding adaptation processes and cultural changes.

The aim of this study was to investigate the morphological diversity of astragali from three modern cattle breeds, one goat breed, and three sheep breeds using three-dimensional GMM methods. This method was chosen for its ability to analyze shape independently of size, making it an ideal tool for uncovering subtle morphological differences between large and small bovids. By applying this method, we aimed to explore how the shape varies between the astragali of cattle (large bovids) and those of sheep and goats (small bovids), providing a comprehensive comparison of their morphological characteristics. Such insights are critical for understanding functional adaptations between these groups. In addition to comparing large and small bovids, this study also evaluated the extent to which the astragalus can differentiate between breeds within species. For cattle and sheep, we tested whether breed-specific morphological patterns exist and whether these differences are significant enough to serve as distinguishing traits. Finally, we compared sheep and goat astragali to identify features that distinguish these closely related small bovids. By focusing on species-specific and breed-specific characteristics, this study contributes to the broader understanding of bovid morphological adaptation and functional diversity. The findings provide critical reference data for ongoing and ancient comparative studies.

## 2. Materials and Methods

### 2.1. Samples

This study was conducted on samples collected between January and July 2024. A total of 225 samples were used in this study. All samples were from 1-year-old male individuals. Only left astragali were collected, and to ensure homogeneity, the samples were selected from the same age and gender group. The samples were obtained from neighboring provinces in Eastern Türkiye. Akkaraman sheep breed (n: 35) samples were collected from the slaughterhouse in Elazığ, Morkaraman sheep breed (n: 38) samples from the slaughterhouse in Diyarbakır, and Hamdani sheep breed (n: 17) and Hair goat breed (n: 50) samples from the slaughterhouse in Siirt. Cattle samples were also collected from the slaughterhouse in Diyarbakır, including 28 Holstein cattle breeds, 22 Hereford cattle breeds, and 35 Simmental cattle breeds.

After being collected from the slaughterhouses, all samples were transferred to the Department of Anatomy, Faculty of Veterinary Medicine, Istanbul University-Cerrahpaşa. The astragali were extracted from the tarsal joints, and boiling was applied. The bones were then immersed in 50% hydrogen peroxide for 4 h to remove adipose tissue and were subsequently dried for further analysis.

### 2.2. Digitalization

The astragali were digitized using a Shining EinScan-SP 3D scanner (Shining 3D, Hangzhou, China) with a camera resolution of 1.3 megapixels (DC 12-volt, 3.33 ampere). A turntable was employed during the scanning process to ensure accurate and consistent capture of all views. A resolution of 1280 × 720 was applied to achieve optimal image quality for scanning the bones. For the processing and integration of the scanned images, the EXScan software (version 3.1.2.0, Shining 3D, Hangzhou, China) was used. This software facilitated the 3D modeling and merging of the individual scan images into a unified model. Once the modeling was completed, the final 3D models of the astragali were saved in PLY file format for further analysis and processing.

**Figure 1:**
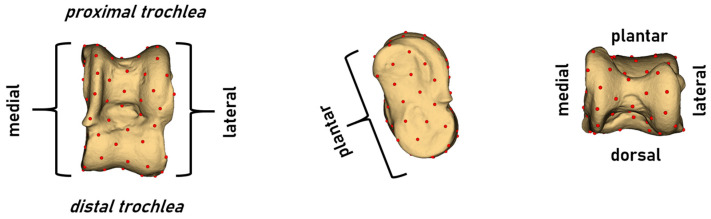
Landmarks and various views of a bovid astragalus.

Due to limited options for anatomical landmarking in the 3D models of the astragali, automatic landmarking was performed for the geometric morphometric (GMM) analysis. This approach allowed for the use of more landmarks, allowing a detailed analysis of the bone structure for comparative purposes. Initially, a draft landmark set was created for this process. The ‘PseudoLMGenerator’ module, part of the Slicermorph add-on in the 3D Slicer software (version 5.5.0), was used to generate a set of 91 equidistant landmarks [15]. This draft set was then applied to all samples through automated landmarking using point cloud alignment and correspondence analysis (ALPACA) [16]. The ALPACA module facilitated the rapid transfer of landmarks from the 3D model and its associated landmark set to target 3D models by utilizing point cloud alignment and shape-deformable mesh registration. As a result of these procedures, a dataset consisting of 91 landmarks for each of the 225 samples was obtained (Figure 1).

### 2.3. Geometric Morphometrics

In this study, 3D raw coordinates were analyzed using RStudio (2024.09.1) software [11]. For the analyses, particularly for shape analysis, the ‘geomorph’ (v.4.0.4) package was utilized [17]. The first step involved standardizing all specimens using Generalized Procrustes Analysis (GPA) [18]. In this process, the size of all specimens was equalized, rotated, and scaled, allowing for clearer isolation of isometry-free shape variation. Thus, the analyses were optimized to reflect only shape differences.

Pairwise comparisons between the squared means of centroid sizes (CSs) for each group were performed to assess size differences among different groups. These comparisons aimed to determine whether the groups significantly differ in size. To evaluate the effect of the size on shape (allometry), which refers to the size-dependent but isometric-free shape component, Procrustes ANOVA was conducted across the entire dataset [19]. This analysis helped us to understand the relationship between shape and size, clearly highlighting the differences in allometric effects among the groups.

The effects of allometric slopes were also analyzed separately. Allometric slopes for large and small bovids were parsed, and separate analyses were conducted for each group. This step enabled us to examine the group-specific differences in the relationship between size and shape, allowing for a comparison of allometric slopes between large and small bovids.

### 2.4. Statistical Analysis

Principal component analysis (PCA) was performed to derive general patterns of morphological variation. Based on the PCA results, the distributions of the shape variation across groups were investigated using the first and the second principal components (PC1 and PC2). To visualize the three-dimensional shape changes for PC1 and PC2, Slicermorph (3D Slicer software, version 5.5.0) was used [15]. A Procrustes ANOVA was then conducted to assess whether the shape differentiation between groups was possible. Additionally, the effect of size on shape (allometry) was evaluated. All analyses were performed using R software [11].

## 3. Results

### 3.1. Shape and Size

The Holstein cattle breed (CS: 334.08) and Simmental cattle breed (CS: 337.56) had larger astragalus sizes compared to the Hereford cattle breed (CS: 307.81) (*p* < 0.001). However, the size difference between Holstein and Simmental breeds was not statistically significant. Similarly, size differences were observed among the small bovid breeds. The Hamdani sheep breed (CS: 135.89) highlighted a larger astragalus compared to the Akkaraman sheep breed (CS: 126.59) and Morkaraman sheep breed (CS: 123.98), whereas the Hair goat breed (CS: 113.60) showed significantly smaller astragalus than the Akkaraman, Morkaraman, and Hamdani sheep breeds (*p* < 0.001). This demonstrates an evident variation in the astragalus size between sheep breeds and goats, suggesting that these differences may be related to morphological adaptation processes. It should provide a concise and precise description of the experimental results, their interpretation, as well as the experimental conclusions that can be drawn.

Significant differences were detected in astragalus shape between large and small bovids (Table 1). These results indicate that astragalus shapes are distinctly different between the two groups. Similarly, significant shape differences were observed between sheep and goat groups, highlighting clear morphological distinctions between these two taxa. In large bovids (Hereford cattle breed, Holstein cattle breed, and Simmental cattle breed), the shape variation between breeds was more pronounced, reflecting a clear morphological divergence. Likewise, among sheep breeds (Akkaraman sheep breed, Hamdani sheep breed, and Morkaraman sheep breed), shape differences were also identified, emphasizing the morphological diversity within the group.

The results of the allometric effect analysis revealed significant differences between large bovids and small bovids. In large bovids, which include the Hereford cattle breed, Holstein cattle breed, and Simmental cattle breed, the relationship between size (centroid size) and shape was found to be notably strong, with 39.6% of the variance explained by size (R^2^ = 0.396, F = 54.367, *p* = 0.001). This result indicates that allometric effects play a prominent and significant role in large bovids. In small bovids, consisting of the Akkaraman sheep breed, Hamdani sheep breed, and Hair goat breed, although the allometric effect was weaker, it was still statistically significant. Here, 13.1% of the variance was explained by size (R^2^ = 0.131, F = 20.782, *p* = 0.001), indicating a more limited influence of size on shape in the case of small bovids. These results suggest that size plays a more dominant role in driving shape variation in large bovids, whereas the effect is weaker and less pronounced in small bovids.

### 3.2. Shape Variation

**Figure 2:**
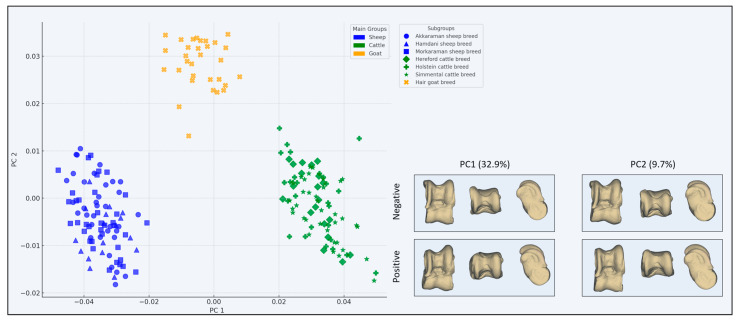
Principal component analysis scatter plot for the bovid astragalus. Models describing how astragalus shape changes between the negative and positive values of PC1 and PC2.

According to the results of PCA, PC1 explains 32.9% of the variance, while PC2 explains 9.7%. This indicates that approximately 42.6% of the total variance is represented by the two main components (Figure 2).

PC1 emerges as the component that explains the most prominent shape variation among the groups, particularly reflecting the differentiation among sheep, cattle, and goat groups. Notably, sheep species are distributed in the negative direction along PC1, while cattle species are distributed in the positive direction. This highlights significant morphological shape variations, especially between the sheep and cattle groups.

Although PC2 accounts for a smaller portion of the variance, it was observed that the goat group is positioned higher with positive PC2 values compared to the other subgroups (sheep and cattle). This indicates that the Hair goat breed morphologically differentiates itself from the other groups.

The PCA results reveal significant morphological variations among the subgroups, providing insight into their distinct structural traits (Figure 2). Within the sheep subgroups, the Akkaraman sheep breed and Morkaraman sheep breed cluster closely along the negative PC1 axis, indicating their morphological similarity, while the Hamdani sheep breed shows slightly greater dispersion, suggesting a broader range of shape variation. In contrast, the cattle subgroups exhibit substantial differences, with the Holstein cattle breed and Simmental cattle breed distributed prominently along the positive PC1 axis. Notably, the Holstein cattle breed demonstrates the highest variance in both PC1 and PC2, reflecting a wider morphological diversity, whereas the Hereford cattle breed remains closer to the origin with lower overall variance, suggesting more uniform morphological traits. The goat subgroup, represented by the Hair goat breed, distinctly separates from the sheep and cattle groups, occupying higher PC2 values, which highlights its unique morphological characteristics.

**Figure 3:**
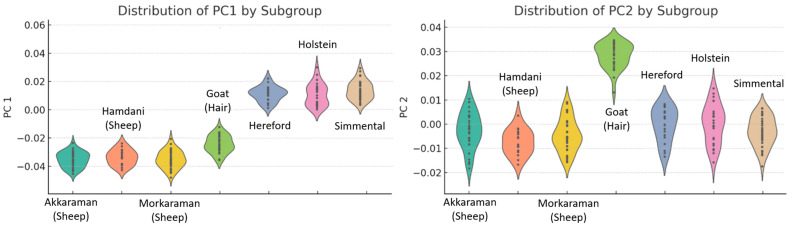
Variations in PC1 and PC2 across subgroups.

Overall, PCA analysis effectively captures and explains the morphological differences at the subgroup level (Figure 3). While the Hair goat breed stands out due to its unique positioning along PC2, the Holstein and Simmental cattle breeds display considerable shape variation along PC1. In contrast, the Akkaraman and Morkaraman sheep breeds reflect tighter clustering, underscoring their morphological similarity. These findings demonstrate the utility of PCA in elucidating group-specific morphological variations and identifying distinct patterns within and across subgroups.

The PCA analysis reveals significant shape variations in the astragalus morphology explained by the PC1 and PC2 components. In PC1, prominent changes are particularly observed in the proximal trochlea. With increasing PC1 values, the lateral portion of the proximal trochlea becomes more proximal compared to the medial portion, while the medial edge appears less developed. In this case, the lateral edge becomes more prominent in positive PC1, whereas the medial edge stands out in negative PC1. Additionally, the astragalus displays a more rounded shape in positive PC1 values, while it adopts a more rectangular form in negative PC1 values.

For instance, in groups with positive PC1 values, such as the Holstein and Simmental cattle breeds, the lateral edge is more developed, and the astragalus exhibits a generally rounded structure. In contrast, in groups with negative PC1 values, such as the Akkaraman and Morkaraman sheep breeds, the medial edge is more prominent, and the astragalus is more rectangular.

In the PC2 component, a different type of shape variation is observed. In positive PC2, while the lateral edge is also well developed, as seen in positive PC1, the shape takes on a more rectangular form. This is exemplified by groups such as the Hair goat breed, where the proximal trochlea shows a developed lateral edge, yet the overall astragalus structure is rectangular. In contrast, in groups with negative PC2 values, such as the Hamdani sheep breed, the astragalus adopts a more rounded structure.

These variations among subgroups effectively highlight the morphological differentiation between species. Specifically, broader and rounded structures are prominent in cattle groups (Holstein and Simmental), narrower and more rectangular forms are observed in sheep groups (Akkaraman and Morkaraman), and the goat group (Hair goat breed) stands out with a developed lateral edge and a rectangular morphology. These findings demonstrate that the astragalus morphology exhibits distinct shape variations across species and subgroups.

## 4. Discussion

This study provides a comprehensive analysis of astragalus morphology in large and small bovids using three-dimensional GMM methods. The findings reveal clear morphological and size-based differences between large bovids (cattle) and small bovids (sheep and goats), as well as significant distinctions within species and between closely related taxa. These results contribute to a deeper understanding of functional adaptations and morphological diversity in bovids, offering information for both modern and ancient comparative studies.

The results demonstrate significant shape differences of the astragali in large and small bovids, showing notable variations in the proximal trochlea. In particular, PC1, which explains 32.9% of the total shape variance, captures key morphological changes related to the lateral and medial edges of the trochlea. In large bovids, such as the Holstein and Simmental cattle breeds, the lateral edge of the proximal trochlea is more developed and more proximally positioned, resulting in a broader and more rounded astragalus. Conversely, in small bovids such as the Akkaraman sheep breed, the medial edge dominates, and the astragalus adopts a more rectangular shape at negative PC1 values. This divergence may reflect functional adaptations to body size and locomotor demands, with large bovids requiring enhanced load-bearing capacity and stability in the lateral side of the astragalus. Similarly, PC2, which explains 9.7% of the total variance, highlights additional morphological changes. While positive PC2 values in the Hair goat breed exhibit a well-developed lateral edge similar to that of large bovids, the overall astragalus structure remains more rectangular. In contrast, negative PC2 values observed in the Hamdani sheep breed are associated with a more rounded astragalus shape, indicating that small bovids exhibit greater variability in proximal trochlea morphology. When the results are evaluated at the species level, the astragalus exhibited distinct morphological characteristics that differentiate *Bos taurus* (cattle), *Capra hircus* (goat), and *Ovis aries* (sheep). In *Bos taurus*, the astragalus is broader and more robust, with a well-developed lateral edge of the proximal trochlea, positioned more proximally. In contrast, *Ovis aries* showed a more rectangular astragalus, where the medial edge of the proximal trochlea is more prominent. *Capra hircus* exhibited a more rectangular astragalus with a well-developed lateral projection. These findings demonstrate that the astragalus exhibits significant shape variations across different bovid species.

Significant morphological and size differences were observed among the cattle breeds. The Holstein and Simmental breeds had significantly larger astragali compared to the Hereford breed. However, the lack of significant size differences between Holstein and Simmental breeds indicates that the shape variation in these groups is less dependent on size. This result aligns with the strong allometric effect observed in large bovids, where 39.6% of the shape variance was explained by size. The prominent role of allometry highlights the importance of mechanical demands and weight distribution in shaping the astragalus morphology of large bovids.

A longer astragalus enhances the power of plantar flexion [6]. This study, by evaluating both large and small bovids, demonstrated that these morphological differences are evident not only in size but also in shape. It was revealed that small bovids possess a thinner and longer astragalus compared to large bovids. This structure suggests that small bovids have greater advantages in terms of agility and mobility compared to their larger counterparts. As Barr also stated, this thin and elongated astragalus form can optimize the dynamic mechanical properties of plantar flexor muscles, enabling more efficient movement in challenging and rugged terrains [6]. In conclusion, our study supports Barr’s findings while also examining the adaptive differences in astragalus morphology between large and small bovids from a broader perspective under different habitat conditions. This demonstrates that morphological variations are not solely limited to size but are also shaped by movement strategies and ecological adaptations.

In the literature, there are morphological and morphometric studies on sheep and goat astragali. For instance, Fernàndez [20] and Haruda [21] reported that sheep astragali are larger than those of goats. Similarly, our study found that the astragalus size of the Hair goat was significantly smaller than that of the examined sheep breeds. This finding suggests that, in adult specimens, the differentiation between goat and sheep astragali can be reliably made based on size alone.

Haruda’s PCA analysis of sheep and goat astragali revealed that specimens with negative values along the first principal component tended to have flatter and more elongated plantar surfaces, resulting in a more rectangular shape [21]. In this distribution, goat specimens were found to occupy negative PC1 values, representing a distinct rectangular morphology. Similarly, Zeder’s study reported comparable patterns; however, it was noted that not all sheep and goat specimens consistently exhibited these features [22]. In our study, the analysis of both PC1 and PC2 variation distributions demonstrated that all goat astragali displayed a more rectangular shape compared to sheep astragali. This finding provides statistical support for the morphological separation between sheep and goat astragali, emphasizing the consistent and well-defined differences between the two species.

The distinct rectangular morphology observed in the goat breed may represent a morphological adaptation for navigating challenging and steep terrains. Compared to sheep, which exhibit a more rounded astragalus and a prominent medial edge, the structural modifications in goats may enhance balance and grip on uneven surfaces, facilitating efficient locomotion in mountainous or rocky environments. However, these interpretations remain morphological predictions. Detailed biomechanical studies are required to determine how these morphological features contribute to locomotion differences between goats and sheep in rugged or mountainous terrains.

In this study, only the left-side astragalus was used, and specimens were collected from animals that were one year old. This approach was specifically chosen to ensure homogeneity and minimize potential sources of variation. The use of a single side of the astragalus reduces asymmetry-related differences, which can naturally occur in paired skeletal elements due to functional loading asymmetry or individual variation [10,23,24]. Standardizing the sample in this way allows for more controlled shape analysis, ensuring that observed differences are due to species- or breed-specific morphological traits rather than lateral asymmetry. While this choice enhances internal consistency within our dataset, it is important to note that some studies may analyze astragali from both sides or focus solely on right-side specimens. Therefore, direct comparisons with studies using bilateral or right-side specimens should consider potential asymmetry effects. Future studies could incorporate both left- and right-side specimens to further assess whether lateral asymmetry influences morphometric findings.

One of the limitations of our study is the inclusion of only a single goat breed, which may not fully represent the morphological diversity within the goat population. Goats can exhibit significant ecological and behavioral differences across breeds, and the morphological adaptations observed in the Hair goat breed may not be generalizable to all goat populations. Additionally, while we analyzed multiple breeds of sheep and cattle, future studies involving a broader range of breeds across all bovid groups would provide a more comprehensive understanding of astragalus variations. However, it is important to note that the sample size in our study was sufficient to ensure the reliability and statistical robustness of the analyses [25]. Expanding the dataset with additional breeds and populations in future research would further strengthen these findings and allow for a deeper exploration of interspecies and intraspecies morphological differences.

## 5. Conclusions

This study highlights the significant morphological differences in the astragalus of large and small bovids, as well as between species and breeds. The findings demonstrate that cattle, sheep, and goats exhibit distinct astragalus shapes, with the cattle astragalus showing a broader and more robust structure, while the same in goats displays a thinner and more rectangular form. Sheep, on the other hand, occupy an intermediate position but tend to have a more rounded morphology of the astragalus compared to goats. This study demonstrated significant morphological differences in the astragalus among species, highlighting its value as a key skeletal element for taxonomic differentiation. The observed shape variations among cattle, sheep, and goats emphasize the taxonomic importance of astragalus morphology in distinguishing closely related species.

Additionally, the morphological differences identified in the astragalus may reflect variations in gait biomechanics, providing insights into the functional adaptations of different bovid species. These findings suggest that future studies focusing on biomechanical analyses could further explore the relationship between astragalus structure and locomotor performance, contributing to a deeper understanding of movement strategies and functional morphology in bovids.

## Figures and Tables

**Table 1:** ANOVA results for shape and size variation in astragalus across bovid groups.

Response Variable	Predictor Variable	Question	R^2^	F	*p*	Interpretation
Shape	Large–Small bovid	Is there a shape difference between large and small bovids?	0.271	68.68	0.001	Yes
Shape	Sheep–Goat	Is there a shape difference between sheep and goats?	0.119	18.618	0.001	Yes
Shape	Cattle breeds	Is there a shape difference among cattle breeds?	0.421	29.869	0.001	Yes
Shape	Sheep breeds	Is there a shape difference among sheep breeds?	0.276	18.873	0.001	Yes
Size	Cattle breeds	Is there a size difference among cattle breeds?	0.453	34.0	<0.001	Yes
Size	Sheep breeds	Is there a size difference among sheep breeds?	0.702	116.7	<0.001	Yes

## Data Availability

The data presented in this study are available upon request from the corresponding author (O.G.).

## References

[1] Dyce K.M., Sack W.O., Wensing C.J.G. (2009). Textbook of Veterinary Anatomy-E-Book.

[2] König H.E., Liebich H.G. (2020). Veterinary Anatomy of Domestic Animals: Textbook and Colour Atlas.

[3] Haruda A.F., Varfolomeev V., Goriachev A., Yermolayeva A., Outram A.K. (2019). A new zooarchaeological application for geometric morphometric methods: Distinguishing *Ovis aries* morphotypes to address connectivity and mobility of prehistoric Central Asian pastoralists. J. Archaeol. Sci..

[4] Popkin P.R., Baker P., Worley F., Payne S., Hammon A. (2012). The Sheep Project (1): Determining skeletal growth, timing of epiphyseal fusion and morphometric variation in unimproved Shetland sheep of known age, sex, castration status and nutrition. J. Archaeol. Sci..

[5] Pöllath N., Schafberg R., Peters J. (2019). Astragalar morphology: Approaching the cultural trajectories of wild and domestic sheep applying Geometric Morphometrics. J. Archaeol. Sci. Rep..

[6] Barr W.A. (2014). Functional morphology of the bovid astragalus in relation to habitat: Controlling phylogenetic signal in ecomorphology. J. Morphol..

[7] Slice D.E. (2007). Geometric morphometrics. Annu. Rev. Anthropol..

[8] Klingenberg C.P. (2016). Size, shape, and form: Concepts of allometry in geometric morphometrics. Dev. Genes Evol..

[9] Boz İ., Altundağ Y., Szara T., Hadziomerovic N., Ince N.G., Pazvant G., Kahvecioğlu O., Özkan E., Manuta N., Gundemir O. (2023). Geometric morphometry in veterinary anatomy. Veterinaria.

[10] Klingenberg C.P. (2015). Analyzing fluctuating asymmetry with geometric morphometrics: Concepts, methods, and applications. Symmetry.

[11] Adams D.C., Otárola-Castillo E. (2013). Geomorph: An r package for the collection and analysis of geometric morphometric shape data. Methods Ecol. Evol..

[12] Mitteroecker P., Gunz P. (2009). Advances in geometric morphometrics. Evol. Biol..

[13] Klingenberg C.P., Monteiro L.R. (2005). Distances and directions in multidimensional shape spaces: Implications for morphometric applications. Syst. Biol..

[14] Colominas L., Evin A., Burch J., Campmajó P., Casas J., Castanyer P., Carreras C., Guardia J., Olesti O., Pons E. (2019). Behind the steps of ancient sheep mobility in Iberia: New insights from a geometric morphometric approach. Archaeol. Anthropol. Sci..

[15] Rolfe S., Pieper S., Porto A., Diamond K., Winchester J., Shan S., Kirveslahti H., Boyer D., Summers A., Maga A.M. (2021). SlicerMorph: An open and extensible platform to retrieve, visualize and analyse 3D morphology. Methods Ecol. Evol..

[16] Porto A., Rolfe S., Maga A.M. (2021). ALPACA: A fast and accurate computer vision approach for automated landmarking of threedimensional biological structures. Methods Ecol. Evol..

[17] Baken E.K., Collyer M.L., Kaliontzopoulou A., Adams D.C. (2021). Geomorph v4. 0 and gmShiny: Enhanced analytics and a new graphical interface for a comprehensive morphometric experience. Methods Ecol. Evol..

[18] Cooke S.B., Terhune C.E. (2015). Form, function, and geometric morphometrics. Anat. Rec..

[19] Cardini A. (2024). A practical, step-by-step, guide to taxonomic comparisons using Procrustes geometric morphometrics and user-friendly software (part A): Introduction and preliminary analyses. Eur. J. Taxon..

[20] Fernàndez H. (2001). Ostéologie Comparée des Petits Ruminants Eurasiatiques Sauvages et Domestiques (Genres Rupicapra, *Ovis, Capra et Capreolus*): Diagnose Différentielle du Squelette Appendiculaire. Doctoral Dissertation.

[21] Haruda A.F. (2017). Separating sheep (*Ovis aries* L.) and goats (*Capra hircus* L.) using geometric morphometric methods: An investigation of Astragalus morphology from late and final Bronze age Central Asian contexts. Int. J. Osteoarchaeol..

[22] Zeder M.A., Lapham H.A. (2010). Assessing the reliability of criteria used to identify postcranial bones in sheep, Ovis, and goats, Capra. J. Archaeol. Sci..

[23] Ocklenburg S., Mundorf A. (2022). Symmetry and asymmetry in biological structures. Proc. Natl. Acad. Sci. USA.

[24] Dongen S.V. (2006). Fluctuating asymmetry and developmental instability in evolutionary biology: Past, present and future. J. Evol. Biol..

[25] Cardini A., Seetah K., Barker G. (2015). How many specimens do I need? Sampling error in geometric morphometrics: Testing the sensitivity of means and variances in simple randomized selection experiments. Zoomorphology.

